# Pilot Evaluation of Intestinal Current Measurement in Cystic Fibrosis and CRMS/CFSPID Patients in Poland

**DOI:** 10.3390/jcm14176020

**Published:** 2025-08-26

**Authors:** Magdalena Postek, Katarzyna Zybert, Lukasz Wozniacki, Marek Woynarowski, Dorota Sands

**Affiliations:** 1 Cystic Fibrosis Department, Institute of Mother and Child, 01-211 Warsaw, Poland; 2 Cystic Fibrosis Centre, Pediatric Hospital, 05-092 Dziekanow Lesny, Poland; 3 Department of Paediatrics, Collegium Medicum, Jan Kochanowski University, 25-317 Kielce, Poland

**Keywords:** cystic fibrosis, ICM, intestinal current measurement, Ussing Chamber

## Abstract

**Background/Objectives:** The term ‘cystic fibrosis transmembrane conductance regulator-related metabolic syndrome/cystic fibrosis screen positive, inconclusive diagnosis (CRMS/CFSPID)’ refers to patients with positive screening tests but without a final diagnosis of Cystic Fibrosis (CF). Intestinal Current Measurement (ICM) is a novel diagnostic technique that may document the abnormal function of the cystic fibrosis transmembrane conductance regulator. Our study aims to compare the cumulative chloride secretory response in the ICM study in the Polish population of CF patients, CRMS/CFSPID, and in a control group. **Methods**: Forceps rectal biopsies were taken from 40 patients (CF; *n* = 17 mean age 9.10 ± 4.18 (0.7–17.20); CRMS/CFSPID: *n* = 16, mean age 6.66 ± 4.83 (0.6–18.0); healthy controls (HC): *n* = 7, mean age 23.7 ± 9.5 (7.8–34.6). ICM tests were performed in the Ussing Chamber according to standard protocol version 2.7 of the European Cystic Fibrosis Society Diagnostic Network Working Group. Delta short circuit-current (ΔIsc) was measured after carbachol (ΔIsc_carbachol_), 3-isobutyl-1-methylxanthine with forskolin (ΔIsc_IBMX/forskolin_), and histamine (Δisc_histamine_) stimulation. Cumulative secretion was calculated for each study group. **Results**: We obtained statistically significant differences in cumulative chloride secretory response between CF and CRMS/CFSPID (CF ΔIsc_carbachol+IBMX/forskolin+histamine_ 15.32 ± 15.47 µA/cm^2^ vs. CRMS/CFSPID ΔIsc_carbachol+IBMX/forskolin+histamine_ 86.84 ± 37.84 µA/cm^2^; *p* < 0.001), and between CF and healthy controls (CF ΔIsc_carbachol+IBMX/forskolin+histamine_ 15.32 ± 15.47 µA/cm^2^ vs. HC ΔIsc_carbachol+IBMX/forskolin+histamine_ 80.16 ± 48.54 µA/cm^2^; *p* = 0.005). No differences in cumulative chloride secretion were observed between the CRMS/CFSPID and HC groups. **Conclusions**: The conducted study suggests that ICM may offer diagnostic value, especially in cases where sweat test results are equivocal.

## 1. Introduction

Cystic Fibrosis (CF) is the most common life-shortening autosomal recessive disorder caused by variants of the cystic fibrosis transmembrane conductance regulator (*CFTR*) gene [1].

Early diagnosis of cystic fibrosis made by the introduction of Cystic Fibrosis Newborn Bloodspot Screening (CF NBS) provides the opportunity to undertake preventive actions and provide treatment before the development of irreversible changes in the respiratory tract and other complications [2]. The pulmonary complications are the leading causes of morbidity and mortality in CF patients. The variable course of the disease progression depends, among others, on the type of *CFTR* variants, hyperinflammation, and pulmonary infection with pathogenic flora [3]. Evidence suggests that respiratory inflammation and infection develop gradually and cause irreversible structural changes in early life [4,5,6,7]. Proper assessment and fast interventions are crucial for delaying and minimizing disease progression. On the other hand, recognition as cystic fibrosis transmembrane conductance regulator-related metabolic syndrome/cystic fibrosis screen positive, inconclusive diagnosis (CRMS/CFSPID) causes stigmatization of patients, which may have a negative impact on their well-being. Therefore, in this group, making a diagnosis of CF or ruling it out is also very important [8,9].

The wide range of *CFTR* variant classes with different intracellular consequences for the underlying defect in the CFTR protein and modifier genes leads to tremendous variability in the clinical phenotype of CF [10,11]. Diagnostic criteria for CF have been established [12] and include guidelines that recommend also assessing CFTR function by alternative methods such as nasal potential difference (NPD) [13,14] and intestinal current measurement (ICM) [14].

In Poland, the CF NBS program covered the entire country from June 2009. The first stage of the protocol is the detection of the elevated immunoreactive trypsinogen (IRT) level in a dried blood specimen taken from the newborn [2,11,15,16]. After a positive IRT result, DNA variants are tested in expanded genetic analysis (IRT/DNA/EGA protocol) [16]. However, the diagnosis of CF may remain incomplete for several reasons, such as ambiguous sweat chloride values, *CFTR* variants of uncertain pathogenicity, and differential expression [11,14,16].

Since 2019, the ICM study has been introduced to the CF Center in Dziekanow Lesny. It is the only facility in Poland that offers ICM testing in the Ussing Chamber system.

The Ussing chamber technique was originally developed by Ussing and Zerahn in 1951 for investigating transepithelial ion transport across amphibian skin [17]. In Cystic fibrosis that technique constitutes a widely employed ex vivo tool for research, facilitating the functional assessment of the transmembrane conductance regulator (CFTR) protein, whose dysfunction underlies the pathophysiology of the disease [18]. This method enables precise evaluation of transepithelial ion transport—particularly chloride and bicarbonate ions—which are essential for fluid homeostasis across epithelial surfaces in various organs [19]. By measuring short-circuit currents and potential differences across epithelial tissues, Ussing chambers provide quantitative insights into CFTR channel activity and the pharmacodynamic effects of CFTR modulators. As such, they play a critical role in both the functional diagnosis of CF and the preclinical development of targeted therapeutics [3,20,21,22]. Two types of micro-Ussing chambers are applied in ICM studies [23]: the re-circulating chamber [24,25] and the continuously perfused chamber [22]. The Rotterdam ICM protocol is optimized for use with the recirculating Ussing chamber, which employs airlifts for oxygenation and fluid recirculation. This system applies voltage clamping to short-circuit the tissue. Advantages of this system include minimal bath volume (1.5–2 mL), preservation of endogenous mediators (e.g., prostaglandins), and precise temperature control. Test compounds are added sequentially and retained throughout the experiment [20]. Multiple variants of the Ussing chamber system are currently available for assessing epithelial permeability, differing in chamber size, bath volume, and exposed tissue area [26]. The short-circuit current (Isc) measured in the Ussing chamber experiments is defined as the net flow of electrical charge per unit time and per unit area of epithelium, under conditions where the transepithelial potential difference (PD_t_) is clamped to zero using an external circuit. Under these conditions, the Isc represents the total transcellular ion transport across the epithelium, as it reflects the net electrogenic movement of ions entering across one epithelial surface and exiting across the opposite surface, thereby completing a closed electrical circuit through the tissue and the voltage-clamp system [23,27].

### Aim

This study aimed to compare delta short-circuit current (ΔIsc) responses among confirmed CF patients, individuals with a questionable CF diagnosis, and healthy controls, and to evaluate CFTR channel function through the use of pharmacological solutions designed to stimulate epithelial ion secretion in rectal tissues.

## 2. Materials and Methods

### 2.1. Ethics

Access to human tissues used in this study was approved by the Bioethics Committee of the Institute of Mother and Child in Warsaw, opinion no.15/2020, approved on 6 April 2020.

A total of 40 individuals—children, adolescents, and adults aged 0.6 to 34 years—were enrolled in this observational study conducted between 2019 and 2021 at the Cystic Fibrosis Center in Dziekanow Lesny, Poland.

The study group included 16 patients with CRSM/CFSPID who were referred for diagnostic confirmation following newborn screening results indicating immunoreactive trypsinogen (IRT) levels above the 99.4th percentile and the identification of two *CFTR* gene variants within the Polish three-tier screening protocol (IRT/DNA/EGA sequencing). These asymptomatic children carried either variants of unknown clinical significance (VUS) or variants of varying clinical consequence (VVCC). Among them, 12 presented with intermediate sweat chloride concentrations, and 4 had normal sweat chloride values accompanied by the detection of a VUS.

The cystic fibrosis (CF) group comprised 17 patients under the regular care of the CF Center in Dziekanów Leśny, including 8 with pancreatic-sufficient CF (PS-CF) and 9 with pancreatic-insufficient CF (PI-CF). The control group consisted of 7 healthy individuals with no history of respiratory or gastrointestinal disease. ICM was measured according to standard operating procedures Version 2.7 26 October 2011 of the European Cystic Fibrosis Society Diagnostic Network Working Group, which was implemented at our CF Electrophysiology Laboratory in October 2019.

Prior to the procedure, each patient received a glycerin suppository to facilitate bowel evacuation. Blood tests were conducted to assess coagulation status and reduce any potential risks related to tissue sampling. A rectal examination was performed to evaluate anatomical suitability and determine eligibility for biopsy.

Rectal biopsies were taken by a trained specialist according to standard procedure using disposable forceps with a diameter of 2.3 mm, without a needle. Measurements were performed on at least 3–4 tissue fragments per subject.

Biopsy samples were immediately placed in ice-cold phosphate-buffered saline (PBS) and transported directly to the ICM technician for dissection and mounting in the Ussing chamber (EasyMount Low Volume Ussing System P2400, Physiologic Instruments, Venice, FL, USA). Sliders (P2407B, Physiologic Instruments, USA) were used to place the tissues in the chamber. The time interval between the start of biopsy collection and the initiation of the Ussing chamber experiment ranged from 10 to 20 min. After approximately 5 min of pre-incubation the basal potential difference (PD basal), short-circuit current (Isc basal) and transepithelial resistance (Rt basal) were measured.

Subsequently, all chambers were preincubated at 37 °C in a buffer solution for 30 min. In the next step the net ion transport current (Isc) across the epithelium was recorded following sequential addition of specific pharmacological agents to the mucosal (M) and/or serosal (S) bathing solutions. The compounds used included amiloride (100 µM), 3-isobutyl-1-methylxanthine (IBMX; 10 µM), forskolin (100 µM), carbachol (100 µM), genistein (50 µM), 4,4′-diisothiocyanostilbene-2,2′-disulfonic acid (DIDS; 200 µM), and histamine (500 µM).

Biopsy responses to secretagogues were included in the analysis only if the tissue was viable, intact, and properly oriented. In accordance with the standard operating procedure (SOP), the minimum acceptable tissue resistance was set at >10.0 Ω·cm^2^.

### 2.2. Statistical Analysis

Data are presented as mean ± standard deviation (SD) and range (minimum–maximum). Statistics were performed with Statistic 13.3. Kruskal–Wallis and Dunn tests with Bonferroni correction were performed for comparison of ΔIsc values in CF, HC, and CRMS/CFSPID. For comparison of ICM between pancreatic sufficient cystic fibrosis patients (PS-CF), pancreatic insufficient cystic fibrosis patients (PI-CF), and ‘CRMS/CFSPID’ groups, the Mann–Whitney U test was used.

## 3. Results

We obtained 150 good-quality biopsies. The obtained tissue samples exhibited resistance values ranging from 10.1 Ω·cm^2^ to 53.0 Ω·cm^2^. Subsequent analyses were performed exclusively on responses to specific stimuli derived from technically valid measurements.

**Figure 1:**
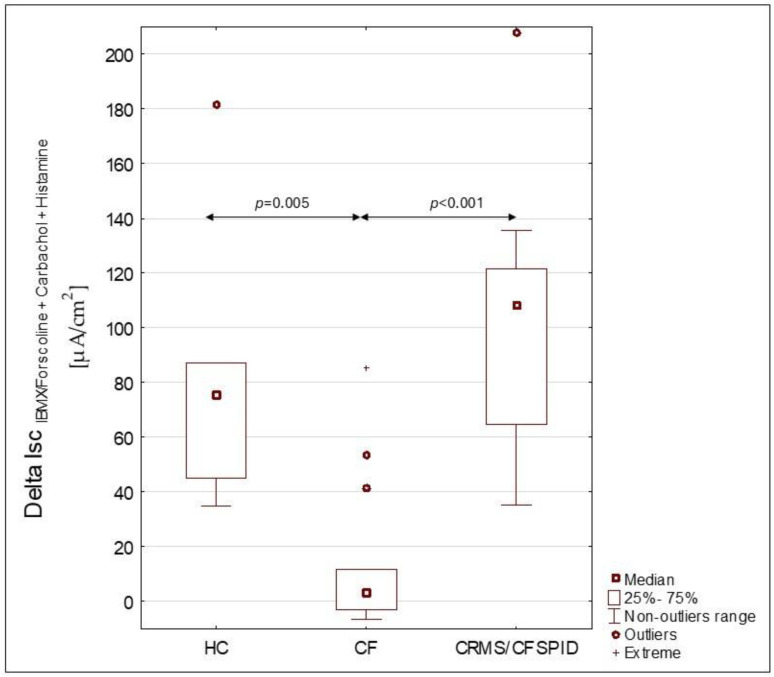
Differences in ΔISC_IBMX/forskolin+Carbachol+Histamine_ for groups HC (healthy controls group), CF (cystic fibrosis patients group) and CRMS/CFSPID (cystic fibrosis transmembrane conductance regulator-related metabolic syndrome/cystic fibrosis screen positive, inconclusive diagnosis patients).

A summary of group-specific characteristics is presented in Table 1. The outcomes of ICM in CRMS/CFSPID *n* = 16, CF *n* = 17 (PS-CF *n* = 8 and PI-CF *n* = 9), and HC *n* = 7 are shown in Table 2. The analysis of signal changes obtained after the use of various secretogenic substances revealed some statistical differences. However, none of the indicators turned out to be a sufficiently sensitive parameter differentiating all groups. We obtained statistically significant differences between Isc_IBMX/forskolin+carbachol+histamine_ in two pairs of groups: CRMS/CFSPID and PI-CF (*p* < 0.001), CRMS/CFSPID and PS-CF (*p* < 0.001). We also obtained a statistically significant difference between PI-CF vs. HC (*p* = 0.02). We did not obtain a significant statistical difference between HC and CRMS/CFSPID and between HC and PS-CF. The results of statistically significant comparisons are shown in Table 2. Figure 1 illustrates the differences observed among the healthy control (HC), cystic fibrosis (CF), and CRMS/CFSPID groups.

## 4. Discussion

ICM is one of the advanced diagnostic methods in cystic fibrosis. It is known that historically ICM reliably discerned the responses of subjects with classic CF and non-CF controls. Unfortunately, for years, there has been substantial variation in reference values between laboratories. This situation changed slightly after the establishment of standard operating procedures (SOPs) by the Therapeutics Development Network (TDN) coordinating center and the European Cystic Fibrosis Society Diagnostic Network Working Group (ECFS DNWG) [9,28]. Nevertheless, many centers are still modifying their protocols; therefore, we decided to conduct a pilot study aimed at establishing our own reference values and comparing the results obtained in the Polish electrophysiology laboratory with published values from other centers.

As we know, patients in whom it is impossible to make a clear diagnosis of cystic fibrosis after a positive NBS result pose a challenge for clinicians both in terms of properly communicating the topic of prognosis to parents and in managing further diagnosis and therapy [29,30]. Moreover, determining the duration of follow-up at a specialized CF center becomes problematic. On the contrary, there is a risk of over-medicalization and stigmatization of children, who may remain with an ambiguous diagnosis for years [31,32].

ICM is a technique that enables the quantitative assessment of CFTR chloride channel function in rectal tissues or other intestinal epithelia [33]. ICM was originally developed to study the CF ion transport defect in the intestine and has been established as a sensitive biomarker of CFTR function and a diagnostic test for CF [28]. It is very important to be able to qualify patients who do not meet diagnostic criteria for cystic fibrosis, while CFTR channel dysfunction has been proven. Among these individuals, a wide range of clinical phenotypes can be found. The factors probably responsible for this variability in phenotype, which have been recognized by clinicians for decades, are the class of variants, the gene modifiers, age at diagnosis, quality and intensity of treatment, and adverse environmental stress [34]. In recent years, significant progress has been made in the evaluation of infants with an equivocal diagnosis after neonatal screening for cystic fibrosis. Electrophysiological tests help to refine the diagnosis in patients with borderline values of chloride in sweat. Compared with lung tissues, rectal biopsies are more easily obtained and thus can be widely used for studies of CFTR channel function. First, rectal epithelium does not express alternative calcium-activated chloride channels [35,36], and, therefore, both cAMP- and calcium-mediated chloride secretion are strictly related to CFTR function. Secondly, the intestine, including the rectum, is not affected by infection or structural organ damage and remodeling [25].

In the Cystic Fibrosis Center Dziekanow Lesny the Intestinal Current measurement tests have been available since 2019. Previously, patients in whom there were indications for in-depth diagnostics and ICM examination were referred to the other European CF Centers. In 2022, the establishment of multicenter cooperation resulted in the publication of data from ICM and NPD tests, including that of Polish children who did not yet have access to this type of diagnostics in the country [37]. The ability to perform the ICM tests in Poland facilitates access to in-depth diagnostics for a larger number of people in need. Especially considering that J.P. Clancy and co-workers showed in their study that CFTR-dependent currents after cold storage for stimulation with forskolin/IBMX, carbachol and forskolin/IBMX + carbachol were, respectively, 44%, 47.5% and 47.3% lower than fresh biopsy [38].

In 2010, N. Derichts and co-workers performed ICM tests in 309 rectal biopsies from 130 infants, children and adults, including patients with known PI-CF (n = 34), PS-CF (n = 7), patients with an unclear diagnosis with mild CF symptoms, intermediate sweat test and/or CFTR variants screening (n = 61) and healthy controls (n = 28). The average responses obtained for HC, PS-CF, PI -CF were, respectively, Isc_Amil_:−5.4 ± 8 μA/cm^2^: −5.5 ± 6 μA/cm^2^; −6.6 ± 7 μA/cm^2^; Isc_cAMP/Forsk_ 16.6 ± 14 μA/cm^2^; 9.8 ± 6; 1.9 ± 2 μA/cm^2^; Isc_Cch_: 36.7 ± 18 μA/cm^2^; 4.2 ± 4 μA/cm^2^; −0.7 ± 7 μA/cm^2^; Isc_His_: 29.3 ± 18 μA/cm^2^; 5.6 ± 5 μA/cm^2^; −2 ± 5 μA/cm^2^. Additionally, they found that the cumulative chloride secretory response of ΔI_sc,carbachol_, ΔI_sc,cAMP/forskolin_ and ΔI_sc,histamine_ was the best diagnostic ICM parameter (cut-off 34 μA/cm^2^) between patients with known PS-CF and controls, differentiating patients with questionable CF into PS-CF (n = 6) and ‘CF unlikely’ (n = 55) groups. We assessed cholinergic and cAMP-CFTR-mediated Cl- secretion in 150 freshly excised rectal biopsies from 40 individuals, including patients with confirmed CF clinical diagnosis (n = 17), individuals with clinical CF suspicion (n = 16) and non-CF controls (n = 7) not in accordance with the original Rotterdam protocol but with the SOP ICM_EU001, V.2.7. As a result of the statistical analysis of discrimination, we obtained that the measurements of Cl-intermediate secretion by CFTR are the best generator differentiating the groups of patients with and without CF. However, we observed lower Isc values than in other centers using the same protocol, for example, R. Minso and co-workers. For 68 healthy controls, they had, respectively, Isc_IBMX//Forsk_ 31 μA/cm^2^ (range 10–104), Isc_Cch_ 77 μA/cm^2^ (range 15–250) and Isc_Histamine_ 72 μA/cm^2^ (range 14–250). The main differences were that they used rectal tissue obtained with a suction biopsy device not forceps like in our laboratory [9]. Also, M. Cohen-Cymberknoh and co-workers found that while the trend in results was comparable, slight variations in the measured values were noted [39]. It is also worth noting the variability observed within the CRMS/CFSPID group. The variability observed in the results may be attributed to the heterogeneity of *CFTR* variants present in the CRMS/CFSPID group, potentially reflecting differences in residual CFTR channel function. This highlights the need to assess the reproducibility of functional responses in relation to specific CFTR genotypes. However, as this was a pilot study, the sample sizes for individual variant combinations were insufficient to allow for meaningful genotype-based comparisons.

Although the numerical results obtained in the Polish laboratory may be lower than in other centers, they do not differ in essence from those obtained in the main world centers. Nevertheless, the introduction of nasal potential differences (NPD), nasal epithelial cells analysis or evaporimetry tests could significantly contribute to the further differentiation of diagnostically difficult cases.

## 5. Study Limitations

This study was designed as a pilot investigation, and several limitations inherent to its exploratory nature should be acknowledged. First, the relatively small sample size—particularly within individual diagnostic subgroups—limits the statistical power of the analyses and precludes robust conclusions regarding potential age-related differences in CFTR function. Although the age range of participants was broad, subgroup comparisons by age were not feasible. Larger, age-stratified studies are needed to address this aspect in more detail. In 2025, Gaerbe et al. reported a study comparing CFTR channel function across different age groups. Their findings demonstrated an age-dependent decline in CFTR-mediated chloride secretion in the rectal epithelium, which was attributed to a reduction in the number of secretory colonocytes located at the base of the colonic crypts [40]. Several recent studies like T. E. Corcoran et al., M. Stahl et al., have highlighted both the progressive decline in CFTR channel function with age and the variability in treatment outcomes with CFTR modulators across different age groups [41,42,43].

Second, the study did not include a comparison between rectal biopsy-based measurements and CFTR function assessed in cultured nasal epithelial cells. At the time of the study, and still currently, our center does not possess the necessary laboratory infrastructure to support nasal epithelial cell isolation culture. However, recognizing the value of this less invasive method, we are actively expanding our laboratory capabilities.

Finally, while rectal biopsy and Ussing chamber analysis represent a well-validated and physiologically relevant method for CFTR functional assessment, the invasive nature of the procedure may limit its routine clinical application, particularly in young children or uncooperative patients.

Despite these limitations, the present pilot study provides important preliminary data supporting the feasibility and diagnostic value of rectal tissue-based CFTR functional assessment in a mixed population, including patients with inconclusive CF diagnoses [28].

## 6. Conclusions

Performing an ICM test on patients with an equivocal result from the screening test for CF may facilitate the diagnostic decision. The conducted studies suggest the usefulness of the ICM test in the differentiation between healthy patients and those with significant dysfunction of the CFTR channel.

## Figures and Tables

**Table 1:** Summarizes the characteristics of the study groups of patients.

	CRMS/CFSPID	HC	CF	PS-CF	PI-CF
N (number of patients)	16	7	17	8	9
Male/Female [%]	7/9 [44/56]	2/5 [29/71]	9/8 [53/47]	4/4 [50/50]	5/4 [56/44]
Age [years]					
Mean ± SD	6.66 ± 4.83	23.7 ± 9.50	9.10 ± 4.18	7.85 ± 4.38	9.04 ± 3.91
Min–max	[0.6–18]	[7.8–34.6]	[0.7–17.2]	[2.5–17.2]	[0.7–15.8]

CRMS/CFSPID—cystic fibrosis transmembrane conductance regulator-related metabolic syndrome/cystic fibrosis screen positive, inconclusive diagnosis patients; HC—healthy controls group; CF—cystic fibrosis patients: PS-CF—cystic fibrosis patients with pancreatic sufficient; PI-CF—cystic fibrosis patients with pancreatic insufficient.

**Table 2:** The outcomes of ICM in groups: CRMS/CFSPID, HC, CF, PS-CF and PI-CF.

ΔIsc response [µA/cm^2^] *	CRMS/CFSPID	HC	CF	PS-CF	PI-CF
Amiloride					
Mean ± SD	−9.71 ± 13.32	−1.15 ± 0.78	−11.03 ± 10.90	−7.18 ± 7.14	−14.46 ± 12.85
[min–max]	[−51.80–2.65]	[−2.70–0.60]	[−37.40–0.30]	[−5.30–20.70]	[−37.40–0.30]
IBMX **/forskolin					
Mean ± SD	28.56 ± 13.95	7.55 ± 5.22	1.65 ± 7.66	5.24 ± 8.49	−1.53 ± 5.48
[min–max]	[8.60–59.30]	[0.00–15.00]	[−13.90–23.90]	[−3.80–23.90]	[−13.90–3.80]
Carbachol					
Mean ± SD	33.04 ± 17.53	36.47 ± 24.04	1.78 ± 6.10	3.28 ± 8.05	0.44 ± 3.68
[min–max]	[−11.50–65.50]	[19.70–88.10]	[−7.30–21.80]	[−3.20–21.80]	[−7.30–5.50]
Histamine					
Mean ± SD	24.64 ± 14.78	36.10 ± 22.72	−0.19 ± 2.50	0.69 ± 2.91	−0.97 ± 1.92
[min–max]	[−10.60–66.40]	[16.80–83.60]	[−4.00–7.40]	[−2.10–7.40]	[−4.00–3.10]
ΔIsc IBMX/Forskolin + Carbachol + Histamine					
Mean ± SD	86.84 ± 37.84	80.16 ± 48.54	15.31 ± 15.47	9.18 ± 18.95	−1.67 ± 10.11
[min–max]	[35.40–166.80]	[43.70–181.40]	[−24.60–53.10]	[−6.50–53.10]	[−24.60–11.90]
*P* values between groups in ΔIsc IBMX/Forskolin + Carbachol + Histamine					
CRMS/CFSPID	n/a	>0.05	<0.001	<0.001	<0.001
HC	>0.05	n/a	0.005	>0.05	0.02
CF	<0.001	0.005	n/a	n/a	n/a
PS-CF	<0.001	>0.05	n/a	n/a	n/a
PI-CF	<0.001	0.02	n/a	n/a	n/a

* ΔIsc—Delta short circuit-current; ** IBMX—3-isobutyl-1-methylxanthine. CRMS/CFSPID—cystic fibrosis transmembrane conductance regulator-related metabolic syndrome/cystic fibrosis screen positive, inconclusive diagnosis patients; HC—healthy controls group; CF—cystic fibrosis patients; PS-CF—cystic fibrosis patients with pancreatic sufficient; PI-CF—cystic fibrosis patients with pancreatic insufficient; n/a—not applicable.

## Data Availability

The data are available from the authors upon request.

## Abbreviations

The following abbreviations are used in this manuscript:

CF	Cystic Fibrosis
CF NBS	Cystic Fibrosis Newborn Bloodspot Screening
CF TDN	Cystic Fibrosis Therapeutics Development Network
CFTR	Cystic fibrosis transmembrane conductance regulator
CRMS/CFSPID	Cystic fibrosis transmembrane conductance regulator-related metabolic syndrome/cystic fibrosis screen positive, inconclusive diagnosis
ECFS DNWG	European Cystic Fibrosis Society Diagnostic Network Working Group
HC	Healthy control
ICM	Intestinal Current Measurement
IRT	Immunoreactive Trypsin
IRT/DNA/EGA	Immunoreactive Trypsin Analysis/Analysis of genetic material, detection of variants in the CFTR gene/Genotypic analysis, identification of variants in the CFTR gene
ISC	Short circuit-current
NPD	Nasal Potential Difference
PBS	Phosphate-Buffered Saline
PI-CF	Cystic fibrosis with pancreatic insufficiency
PS-CF	Cystic fibrosis with pancreatic sufficiency
SOP	Standard operating procedure
VUC	Variant of unknown clinical significance
VVCC	Variant with varying clinical consequence
